# The devil is in the details: Variable impacts of season, BMI, sampling site temperature, and presence of insects on the post-mortem microbiome

**DOI:** 10.3389/fmicb.2022.1064904

**Published:** 2022-12-07

**Authors:** Aaron M. Tarone, Allison E. Mann, Yan Zhang, Roxanne R. Zascavage, Elizabeth A. Mitchell, Edgar Morales, Travis W. Rusch, Michael S. Allen

**Affiliations:** ^1^Department of Entomology, Texas A&M University, College Station, TX, United States; ^2^Department of Microbiology, Immunology, and Genetics, University of North Texas Health Science Center, Fort Worth, TX, United States; ^3^Department of Biological Sciences, Clemson University, Clemson, SC, United States; ^4^Center for Grain and Animal Health Research, USDA Agricultural Research Service, Manhattan, KS, United States

**Keywords:** post-mortem microbiome, metataxonomics, forensic microbiology, decomposition, necrobiome

## Abstract

**Background:**

Post-mortem microbial communities are increasingly investigated as proxy evidence for a variety of factors of interest in forensic science. The reported predictive power of the microbial community to determine aspects of the individual’s post-mortem history (e.g., the post-mortem interval) varies substantially among published research. This observed variation is partially driven by the local environment or the individual themselves. In the current study, we investigated the impact of BMI, sex, insect activity, season, repeat sampling, decomposition time, and temperature on the microbial community sampled from donated human remains in San Marcos, TX using a high-throughput gene-fragment metabarcoding approach.

**Materials and methods:**

In the current study, we investigated the impact of BMI, sex, insect activity, season, repeat sampling, decomposition time, and temperature on the microbial community sampled from donated human remains in San Marcos, TX using a high-throughput gene-fragment metabarcoding approach.

**Results:**

We found that season, temperature at the sampling site, BMI, and sex had a significant effect on the post-mortem microbiome, the presence of insects has a homogenizing influence on the total bacterial community, and that community consistency from repeat sampling decreases as the decomposition process progresses. Moreover, we demonstrate the importance of temperature at the site of sampling on the abundance of important diagnostic taxa.

**Conclusion:**

The results of this study suggest that while the bacterial community or specific bacterial species may prove to be useful for forensic applications, a clearer understanding of the mechanisms underpinning microbial decomposition will greatly increase the utility of microbial evidence in forensic casework.

## Introduction

Post-mortem microbiomes have been studied intensively since the advent of next generation sequencing in the context of forensic science, medicine, and decomposition ecology since they are informative of a variety of factors associated with death investigations, the physiological states of the previously living, and ecosystem functions (Metcalf et al., 2013; Pechal et al., 2014; Cobaugh et al., 2015; Singh et al., 2018; Kaszubinski et al., 2020). There is experimental evidence indicating that microbial data can provide information associated with post-mortem intervals (PMI), locations of death, and causes of death (Can et al., 2014; Finley et al., 2015; Metcalf et al., 2016; Pechal et al., 2018; Zhang et al., 2019). In post-mortem microbiome work, the general concept is that a decedent is a microbial ecosystem that will undergo a predictable succession of community members over time. Across experiments, certain taxa at the phyletic (e.g., Gammaproteobacteria, Bacteroidetes, Actinobacteria; Pechal et al., 2013; Adserias-Garriga et al., 2017), family (e.g., Moraxellaceae, Enterobacteriaceae, Planococcaceae; Pechal et al., 2014), or generic (e.g., *Proteus*, *Ignatzschineria*, *Clostridia*; Metcalf et al., 2016) levels occur in different abundances depending on whether samples are collected early or later in the decomposition process.

While there is a general indication that there are reliable temporal signals in microbiome community data, it is also clear that more needs to be understood before microbiomes are ready for forensic applications (Metcalf, 2019). For example, some post-mortem microbiome studies indicate the ability to predict the post-mortem interval (PMI) with approximately 90% accuracy (Belk et al., 2018), while others predict approximately 71% accuracy (Pechal et al., 2018; Zhang et al., 2019). Some of the differences between such experiments could be due to sample size, differences in regions (e.g., temperature or presence of pollutants), or related to experimental design and methodologies. However, ecological factors like seasonal impacts on post-mortem microbial communities are also demonstrated (Carter et al., 2015). This observation is important because it implies the need for seasonally (and other) adjusted predictions with microbiome data. However, while seasonal effects are evident, a mechanistic understanding of their underpinnings remains to be fully appreciated.

**Figure 1:**
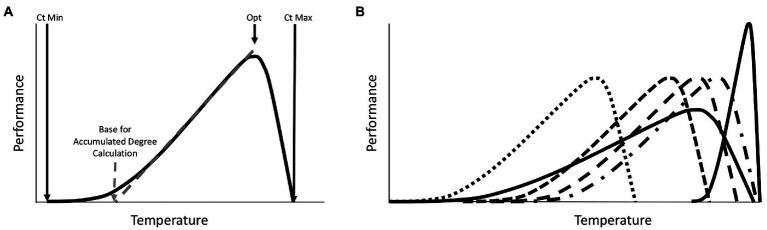
Expectations for a general phenotypic performance response to temperature for one species. **(A)** All phenotypes for all organisms exhibit a thermal performance range defined by the Critical Thermal Minimum (Ct Min) and Critical Thermal Maximum (Ct Max). Responses are typically skewed such that optimal performance is closer to Ct Max than Ct Min. Changes in performance with increasing temperature are usually shallower at lower temperatures and steeper at higher temperatures. For some portion of the thermal performance curve the response to temperature is relatively linear and can be predicted with simple linear accumulated degree models. The base temperature for such models (below which development is assumed to be zero) is defined by the intercept of this linear response with the temperature axis at the point where performance is zero. This linear range is a subset of the full thermal performance curve. Outside of this range, thermal predictions can be biased if linear models are applied. Organisms often regulate their temperatures by orienting close to, but usually not exactly, their optimal temperatures (Opt), which can vary across phenotypes, environments, and genotypes studied. **(B)** A theoretical distribution of thermal performance for a community of organisms, where each species has distinct thermal performance curves, with different thermal minima, maxima, and optima, which reveals patterns for thermal specialist or generalist, and where some species may or may not co-occur due to differences in thermal performance. The members of the community have different optima, different base temperatures for accumulated degree calculations, and different thermal ranges are covered by the linear portions of the thermal responses of individual community members. Such community members may be informative of an ecological process in different thermal conditions, but imposing a single thermal assumption on a whole community of organisms is more problematic than predictions of one well understood organism.

One obvious explanation for seasonal variation in microbiomes is that seasons differ in temperature. This is a special variable, in that it is the only one that is regularly accounted for in current approaches to studying post-mortem microbiomes. However, even in this instance of clear importance, there is room for improvement when making forensic estimates with post-mortem microbiome community data. Most forensic prediction studies adjust for temperature-specific growth rates by applying accumulated degree models to community data. Usually these are derived from a simple assumption that microbial growth generally does not occur below 0°C and that the response to temperature increases is linear (Pechal et al., 2014; Belk et al., 2018). However, basic thermal biology makes it clear that this is not expected to be the case for any group of organisms (Angilletta, 2009). Organismal performance exists within a range of operable temperatures. The response is curvilinear, with an optimal temperature that typically is closer to the critical thermal maximum than the critical thermal minimum. Most importantly, the range of temperatures that can be survived, and the temperatures at which a linear relationship between temperature and performance exist, are phenotype and organism (and even sometimes genotype) dependent (Figure 1). Thus, while estimates are made with communities of microorganisms, the thermal assumptions about these organisms are not equally valid across all members of the community. There is a good reason why this practice of assuming the same base temperature for an entire community exists - there is currently no better set of assumptions that can be applied to a community of bacteria for this purpose. However, basic thermal biology can explain seasonal effects on predictions, and predicts that certain temperatures may be more prone to inaccuracy than others when a specific reference data set (collected at a specific temperature range) is applied. While temperature is clearly an important factor in estimates with microbiomes, other seasonally variable factors could also impact the results (Singh et al., 2018).

In addition to the fact that thermal variation may impact post-mortem microbiomes, there are also demonstrated impacts of insect colonization (Pechal et al., 2013; Iancu et al., 2015, 2020; Tomberlin et al., 2017), which themselves can differ across seasons. For example, *Ignatzschineria* is a known marker taxon during early decomposition (Metcalf et al., 2016). However, it is also known to be associated with insect colonization. In the absence of this taxon, other genera, such as *Proteus* proliferate in the same time frame (Tomberlin et al., 2017). Recently, the genus has been suggested as a marker of insect colonization of remains (Iancu et al., 2020). Carrion feeding insects are known to alter their microbiomes through their own excretions and secretions (Sherman et al., 2000; Cazander et al., 2009; Barnes et al., 2010), which include immune peptides (Nygaard et al., 2012), symbiont microbes (Shukla et al., 2018), and microbial products (Erdmann and Khalil, 1986; Sherman et al., 2000). For example, the carrion fly *Lucilia sericata* exhibits an expanded repertoire of antimicrobial peptides compared to *Drosophila* genomes (Pöppel et al., 2015). This fly expresses distinct antimicrobial peptide profiles in the presence of different bacteria (McKenna et al., 2022). Further, blow fly associated *Proteus mirabilis* make phenolic compounds dubbed “mirabilicides” that are toxic to other microbes (Erdmann and Khalil, 1986). Thus, it is not surprising that the presence of *Proteus mirabilis* in fly guts has been shown to alter levels of *Salmonella enterica* serovar Typhimurium (Greenberg, 1969). Insect communities change throughout the year (Tomberlin and Adler, 1998; Cammack et al., 2016), thus seasonal communities could impact seasonal microbiomes on remains if the differing taxa interact with microbes differently. Finally, other individual-specific factors such as body mass index (BMI) and biological sex may also shape the microbes involved in decomposition (Pechal et al., 2018; Singh et al., 2018; Zhang et al., 2019). For example, Singh et al. (2018) noted that some Gammaproteobacteria signatures in soil under remains was positively correlated with cadaver starting mass.

To address whether post-mortem microbiome seasonality is due to insects, temperature, or other factors, we evaluated microbiomes from donated human remains that had decomposed for seven to 272 days in San Marcos, TX. During collections, we recorded BMI and sex of the deceased from associated donation data, if insects were present at swabbing sites, and recorded temperatures of collection sites to determine if these factors impacted the microbiomes of decomposing remains and the temporal signals associated with them.

## Materials and methods

### Experimental design

We collected nostril swabs from twenty deceased individuals located at the Forensic Anthropology Center, Texas State University, San Marcos, TX during two seasons. Ten individuals each were sampled during the summer (July 19, 2019) and winter (February 26, 2019). Each individual was swabbed four times to document intraindividual variation for a total of 80 swabs plus an additional two air-exposed blank control swabs per sampling season. Surface temperatures at the swab site (in the nostril) were collected at the time of sampling using a thermal camera (model T650sc ©Teledyne FLIR, USA) and ranged from 24.5°C to 54.5°C with an average sample site surface temperature during the summer of 43.2°C (SD ± 4.2°C) and an average sample site surface temperature in the winter of 31.2°C (SD ± 3.8°C). To test the impact of different lysing matrices, 30 swabs were lysed with Lysing Matrix A and 29 with Lysing Matrix E (MP Biomedicals, Irvine, CA). Along with sampling site temperature, the presence of insects at the sampling site (within the nostril), presence on the body but not in the nostrils, or absence of insect activity was recorded for each individual. Insect activity at the sampling site was found in 12 individuals sampled in the summer, and 12 individuals sampled in the winter. Additionally, the number of days since an individual had been introduced to the field was recorded. Ranging from seven to 272 days, the average time in the field for an individual was 80 days (SD ± 91 days). Finally, Body Mass Index (BMI) and sex of each individual was recorded. Full metadata for all samples can be found in Supplementary Table 1.

### DNA extraction and library preparation

DNA was extracted from 40 summer and 40 winter nasal swabs and blanks using the MPBio FastDNA™ SPIN Kit for Soil (MP Biomedical, Irvine, CA). The swab heads were aseptically transferred to lysing matrix bead tubes and snapped from their handles. Two of the four samples taken from each individual were processed using Lysing Matrix A, and the remaining two were processed using Lysing Matrix E following the MPBio FastDNA protocol including homogenization on an MPBio FastPrep instrument (6 m/s, 40 s). Extractions were performed as outlined by the manufacturer. DNA was eluted into clean, 1.5 ml microcentrifuge tubes in DNase free water, quantified using a Qubit dsDNA HS Assay Kit (Invitrogen, Carlsbad, CA), and stored at −20°C until further processing.

The Illumina protocol for 16S rRNA Metagenomic Sequencing Library Preparation (Part # 15044223 Rev. B) was used for amplification of the 16S rRNA gene’s V4 region using the 515F (5′-GTGYCAGCMGCCGCGGTAA-3′) and 806R (5′-GGACTACNVGGGTWTCTAAT-3′) primer set. PCR amplification was conducted on all samples in duplicate, however only 26 out of 40 winter swabs and 34 out of 40 summer swabs were band positive. A positive control of *E. coli* genomic DNA was included in batches as a PCR amplification control. No template controls consisting of molecular grade water were also included throughout the amplification process and sequenced to assess possible contamination. Duplicate positive PCR products from each sample were combined prior to post-PCR clean-up using AMPure magnetic beads. After cleanup, the PCR products were indexed using Illumina Nextera XT Index Kit v2 (Illumina, San Diego, CA) following the manufacturer’s instructions and purified again using AMPure XP magnetic beads (Beckman Coulter, Chaska, MN). Quantification of indexed PCR products was performed using the Qubit dsDNA HS Assay Kit (Invitrogen, Carlsbad, CA). To avoid sampling bias, the samples were separated at random into two separate sequencing runs containing a combination of the summer and winter samples. Furthermore, the samples chosen had to contain one of each of the lysing matrices used for DNA extraction to further reduce any sampling bias. The libraries were sequenced using the Miseq Reagent V2 (500 cycle) kit. After sequencing and processing, an additional two winter sample swabs were removed following QC of the sequencing data, leaving 34 summer swabs from 10 remains, and 24 winter swabs from 8 remains, for a total of 58 samples analyzed here. Each set of remains was covered by at least 2 swabs. Further details can be found in Supplementary Table 1.

### Quantitative PCR analysis

To determine if shifts in the proportion of different major bacterial taxa could be used to estimate the PMI, we performed qPCR analysis on a subset of individuals using taxon-specific primer sets for the phyla Actinobacteria, Firmicutes, Bacteroidetes, and the order Gammaproteobacteria. A total of 18 individuals were included (10 summer samples and 8 winter samples). Individuals included in this analysis are indicated with an asterisk in Supplementary Table 1. We performed qPCR on these samples on an Applied Biosystems QuantStudio 5 instrument (Waltham, MA) using a SYBR Green (Applied Biosystems, Waltham, MA) assay for absolute quantification of all samples following modification of a previously published protocol (Yang et al., 2015). Briefly, samples were run in duplicate on four plates with each plate used to target a single phylum. qPCR was prepared using 12.5 μl of 2X SYBR master mix, 0.5 μl each of forward and reverse primers (200 nM final concentration), 9 μl of water, and 2.5 μl of sample. Run parameters were 95°C for 20 s followed by 60°C for 1 min and repeated for 40 cycles. Raw results were compared to a standard curve to determine copy number. Seven point standard curves from 10^2^–10^8^ copies were generated using genomic DNA template from appropriate bacteria purchased from ATCC (Actinobacteria, *Cutibacterium acnes* ATCC 11828D-5; Bacteroides, *Bacteroides vlugatus* ATCC 8482D-5; Betaproteobacteria, *Neisseria meningitides* ATCC 700532D-5), or prepared in-house from bacterial culture (Gammaproteobacteria, *Aliivibrio fischeri* ATCC 7744; Firmicutes, *Streptococcus pneumoniae* ATCC 6308, courtesy of Dr. Harlan Jones). Mean results were evaluated for trends that could be further evaluated in determining PMI.

### Computational methods

DNA sequences were processed as previously described (Mann et al., 2020). Briefly, we first removed primers from the raw sequence data using Cutadapt (v.2.10; Martin, 2011). Next, we quality filtered, dereplicated, merged paired end reads, removed chimeras, and generated amplicon sequence variants (ASVs) using the DADA2 pipeline (v.1.14.1; Callahan et al., 2016). Samples with fewer than 10,000 reads post quality filtering were removed from downstream analysis. Post-filtering, we retained 58 high-quality samples and generated 2,989 unique ASVs (Supplementary Table 2). We assigned a taxonomy to each ASV using VSEARCH (v.2.8.1; Rognes et al., 2016) as implemented in QIIME2 (2020.8; Bolyen et al., 2019). Any ASVs that could not be assigned a taxonomy or were assigned only to the kingdom level (i.e., Bacteria) were removed from downstream analyzes. A representative tree of our filtered ASVs was generated using FastTree (v.2.1.10; Price et al., 2010). All analyzes and figure generation was performed in R (v.3.6.1; R Core Team, 2017). Beta diversity metrics were performed on PhILR (Silverman et al., 2017) transformed data and visualized as a hierarchical cluster dendrogram using ggplot2 (Wickham, 2016) or PCA using the auto plot function in ggbio (Yin et al., 2012). To determine which taxa might account for the observed variation in our beta diversity plots using a different metric we also generated a NMDS biplot based on a Bray–Curtis dissimilarity matrix using phyloseq (McMurdie and Holmes, 2013). We next calculated the impact of season, sampling site temperature, insect presence, BMI, and sex on the microbial community with PERMANOVA using the adonis function in vegan (v.2.5-6; Oksanen et al., 2019). Finally, we determined the predictive power of different metadata categories on the total microbial community using a random forest classification model with 10,000 trees or regression model with 500 trees with the randomForest (v.4.6–14) and rfUtilities (v.2.1-5; Liaw and Wiener, 2002) packages. Conda environment and all processing and analysis scripts are provided at https://github.com/aemann01/necrobiome and are archived on Zenodo (DOI: 10.5281/zenodo.5722411) for analytical reproducibility.

## Results

**Figure 2:**
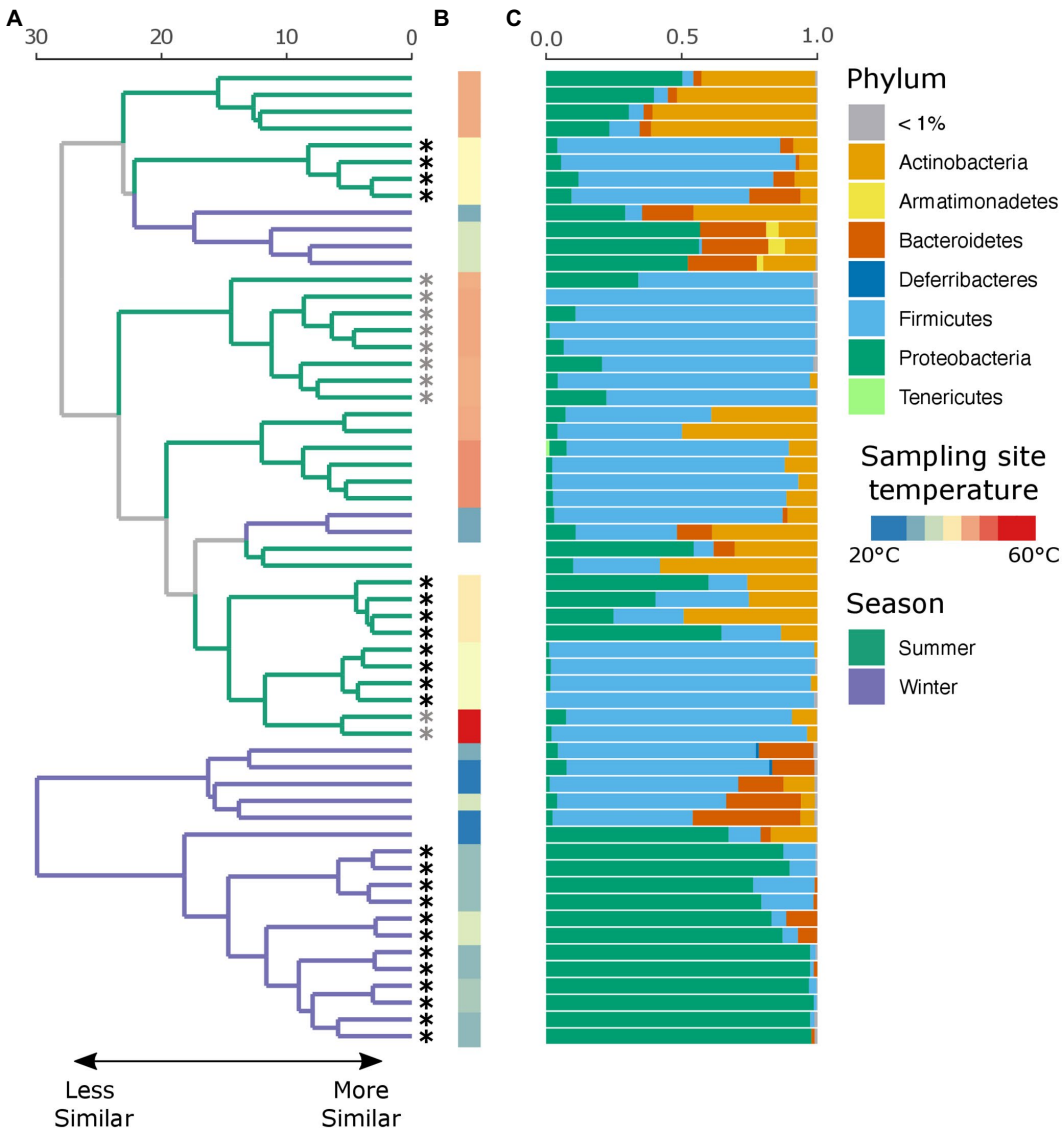
Local clustering of samples by season, surface temperature, and presence of insects. **(A)** Hierarchical cluster dendrogram of PhILR transformed beta diversity. Each branch tip represents a single sample. Colors on branches indicate whether the sample was collected in the summer (green) or winter (purple). Asterisks (*) at tips indicate that insects were present at the time of sampling. Gray asterisks denote samples where insects were present but not at the sampling site (nostril). Black asterisks indicate samples where insects were in the nares. **(B)** Surface temperature at time of sampling as measured by thermal imaging corresponding. Two samples had no recorded temperature and were thus left blank. **(C)** Relative abundance of taxa at the phylum level for each sample.

An average of 134,152 (SD ± 66,338) reads were recovered across all samples post quality and chimera filtering (Supplementary Table 3). No reads in the two negative controls passed quality filtering steps and were removed from downstream analysis. Very few ASVs were shared across the winter and summer samples with 78 and 79% of all detected ASVs unique to that seasonal group, respectively (Supplementary Figure 1A). Moreover, individuals with no insect activity had the highest proportion of unique ASVs as compared to those with insect activity (Supplementary Figure 1B), reflecting the dominance of insect-associated bacteria in affected individuals. We found that season, insect activity, sex, BMI, and the temperature at the site of sampling were substantial factors in shaping the retrieved microbial community. Samples collected in the summer tended to be dominated by Firmicutes, and in particular *Clostridium* sp., while samples collected in the winter tended to be dominated by either Firmicutes or Proteobacteria, the proportion of which largely depends on insect activity (Figure 2C). If insects were present, the samples were predominantly composed of bacteria belonging to the *Ignatzschineria* genus. In fact, samples with insect activity had higher levels of Proteobacteria, independent of season or surface temperature, though this effect is more apparent in the winter samples, likely due to lower starting microbial diversity. BMI correlated with *Corynebacterium*, *Enterococcus*, and *Clostridium*.

While there were no significant differences in alpha diversity between winter and summer samples as measured by both the number of observed ASVs and Shannon diversity (*p* = 0.99, Supplementary Figure 2), season was a significant factor in shaping the full microbial community by PERMANOVA test (*p* = 0.001, *R*^2^ = 0.18), as was insect presence (*p* = 0.001, *R*^2^ = 0.23), surface temperature (*p* = 0.001, *R*^2^ = 0.27), sex (*p* = 0.002, *R*^2^ = 0.09), and decedent BMI (*p* = 0.001, *R*^2^ = 0.21). The lysing matrix used had no significant impact on the detected microbial community (*p* = 0.98). As surface temperature and presence of insects is correlated with seasonality, we also tested the impact of insects and temperature within season. Insect presence was a significant factor in shaping the microbial community in both winter (*p* = 0.001, *R*^2^ = 0.49) and summer (*p* = 0.002, *R*^2^ = 0.17) as was surface temperature (winter: *p* = 0.011, *R*^2^ = 0.19; summer: *p* = 0.001, *R*^2^ = 0.25). A random forest classification model successfully identified summer samples in 94.12% of cases and winter samples in 91.67% of cases. The top discriminant taxa between the seasons include two *Clostridium* spp. (ASV5, ASV10), *Sporosarcina* sp. (ASV2), *Corynebacterium* sp. (ASV4), and *Ignatzschineria* sp. (ASV29). Insect presence at the sampling site was successfully identified in 100% of cases while the absence of insects was successfully identified in 87.50% of cases. Interestingly, the presence of insects elsewhere on the body but not at the sampling site was successfully identified in 80% of cases with the remaining 20% of cases misclassified as samples with insects at the sampling site. The top taxa that discriminate between the presence or absence of insects were *Ignatzschineria* sp. (ASV3), *Providencia* sp. (ASV103), two *Clostridium* spp. (ASV34, ASV57), and *Lactobacillus* sp. (ASV71). A hierarchical clustering dendrogram of beta diversity metrics across samples illustrates the impact of season, insect activity, and surface temperature on the microbial community (Figures 2A,B). *Ignatzschineria*, *Providencia*, *Clostridium*, and *Lactobacillus* are known to be associated with blow flies (Tomberlin et al., 2017).

**Figure 3:**
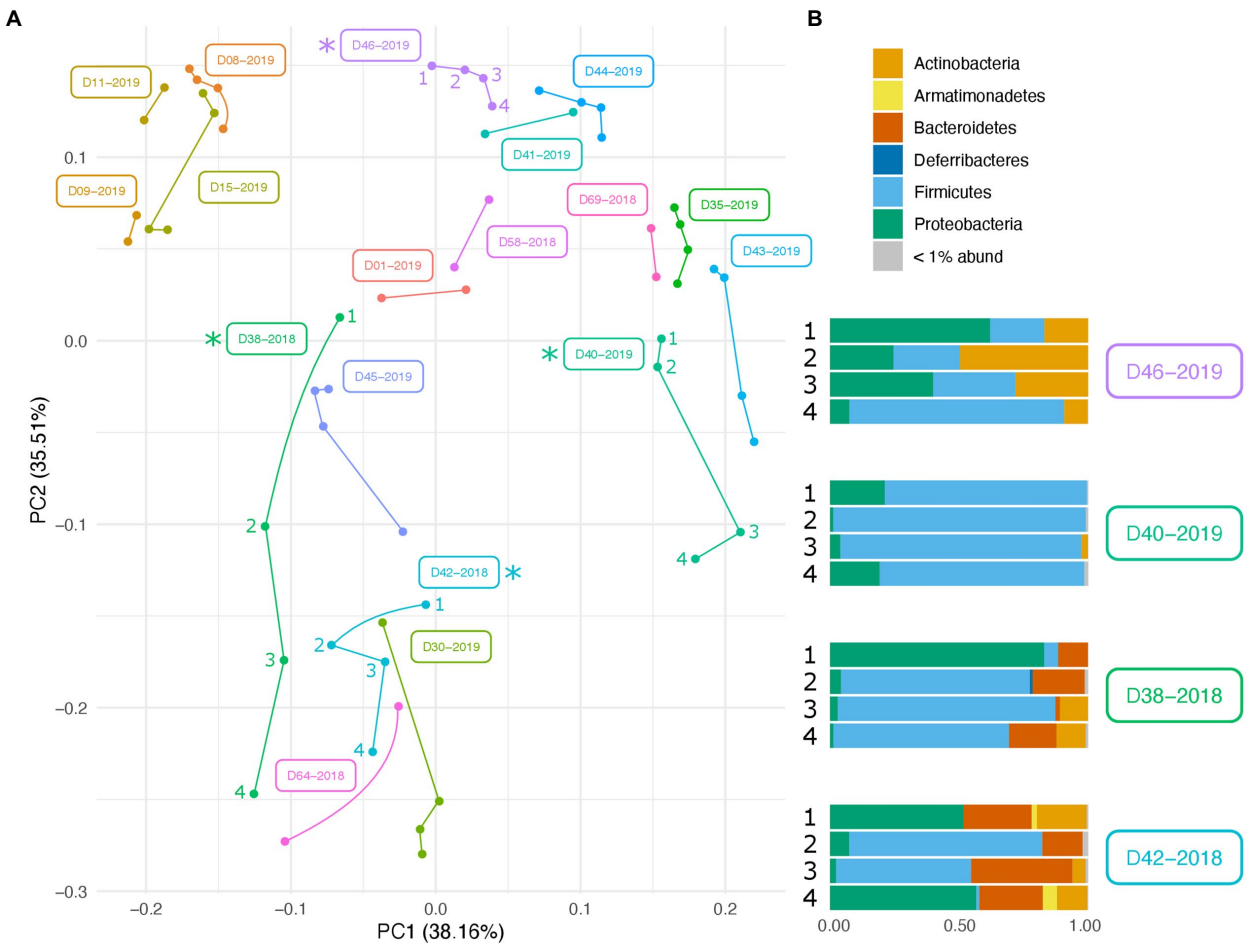
Variation in microbial community across swabs taken from the same individual. **(A)** PCA plot of PhILR transformed beta diversity metrics. Individual swabs are represented as points. Swabs taken from the same individual are connected by a line. **(B)** Taxonomic composition differences across swabs taken from the same individual at the same sampling point. Corresponding samples are indicated on the PCA plot with an asterisk.

**Figure 4:**
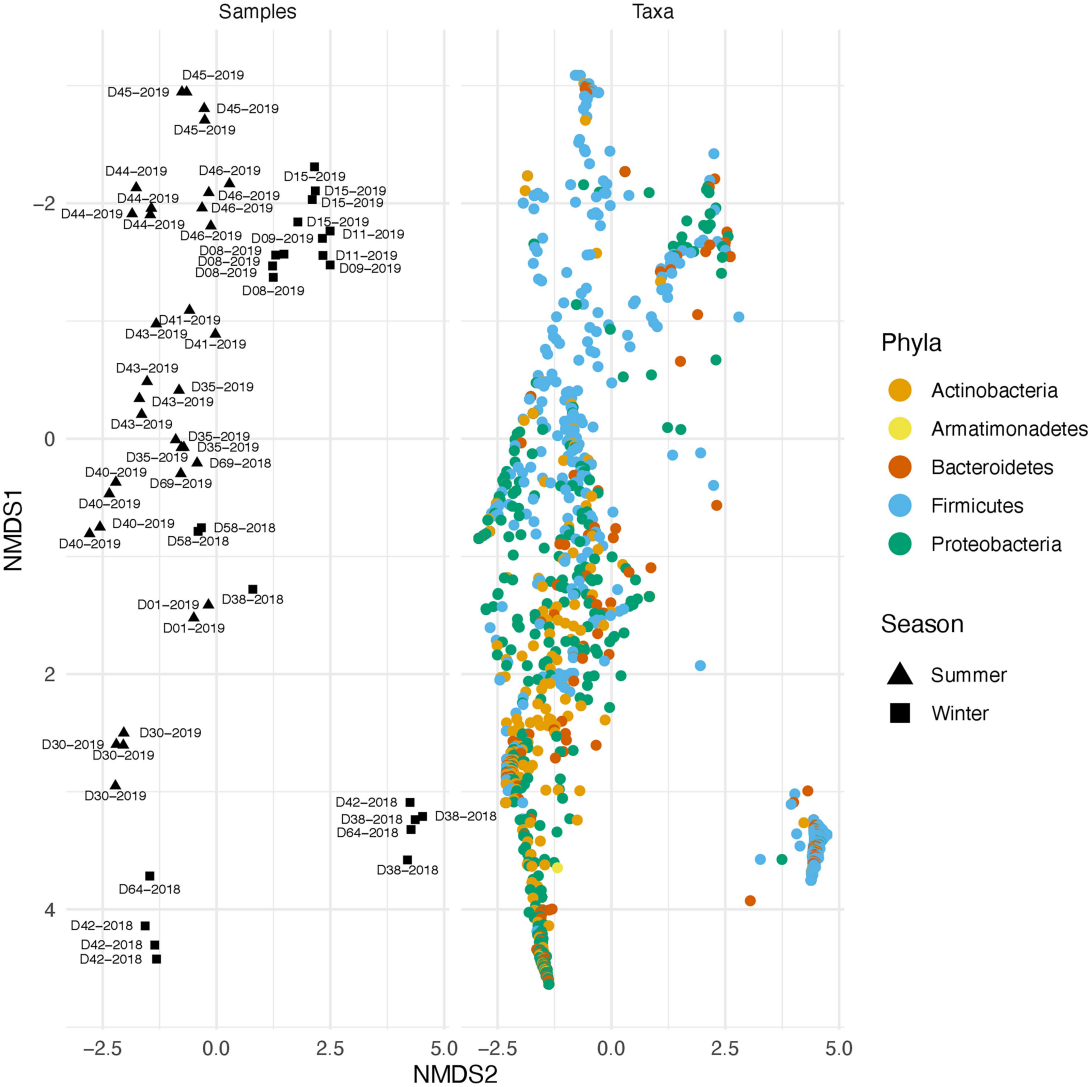
Taxonomic shift across different samples in summer and winter. Variation across samples is primarily driven by a shift in specific phyla. The plot on the right illustrates beta diversity across individual swabs. Plot on the left is identical to the one on the right but instead of samples, ASVs colored by phylum are plotted.

Along with environmental effects on the microbial community, we found that duplicate swabs from the same individual may in some cases produce markedly different results (Figure 3). This effect is moderately correlated with the length of time that an individual has been deposited in the field, with longer exposed individuals having higher maximum distances between swabs (*R*^2^ = 0.65, *p* = 0.004; Supplementary Figure 3). The highest maximum distance between swabs collected from the same nare of the same individual was 26.04 (D38-2018) and the lowest at 2.39 (D09-2019; Supplementary Table 4). A biplot of phyla importance along the y axis of a NMDS plot illustrates that much of the observed variation across individual swabs is due to a compositional shift from Firmicutes to Proteobacteria and Actinobacteria (Figure 4). Nares of older remains tended to be drier and results could reflect differences in surface and subsurface taxa that were removed sequentially among replicate swabs in this situation. Whereas younger remains were often full of maggots in what amounted to an aqueous environment where microbes would be expected to be more evenly mixed.

**Figure 5:**
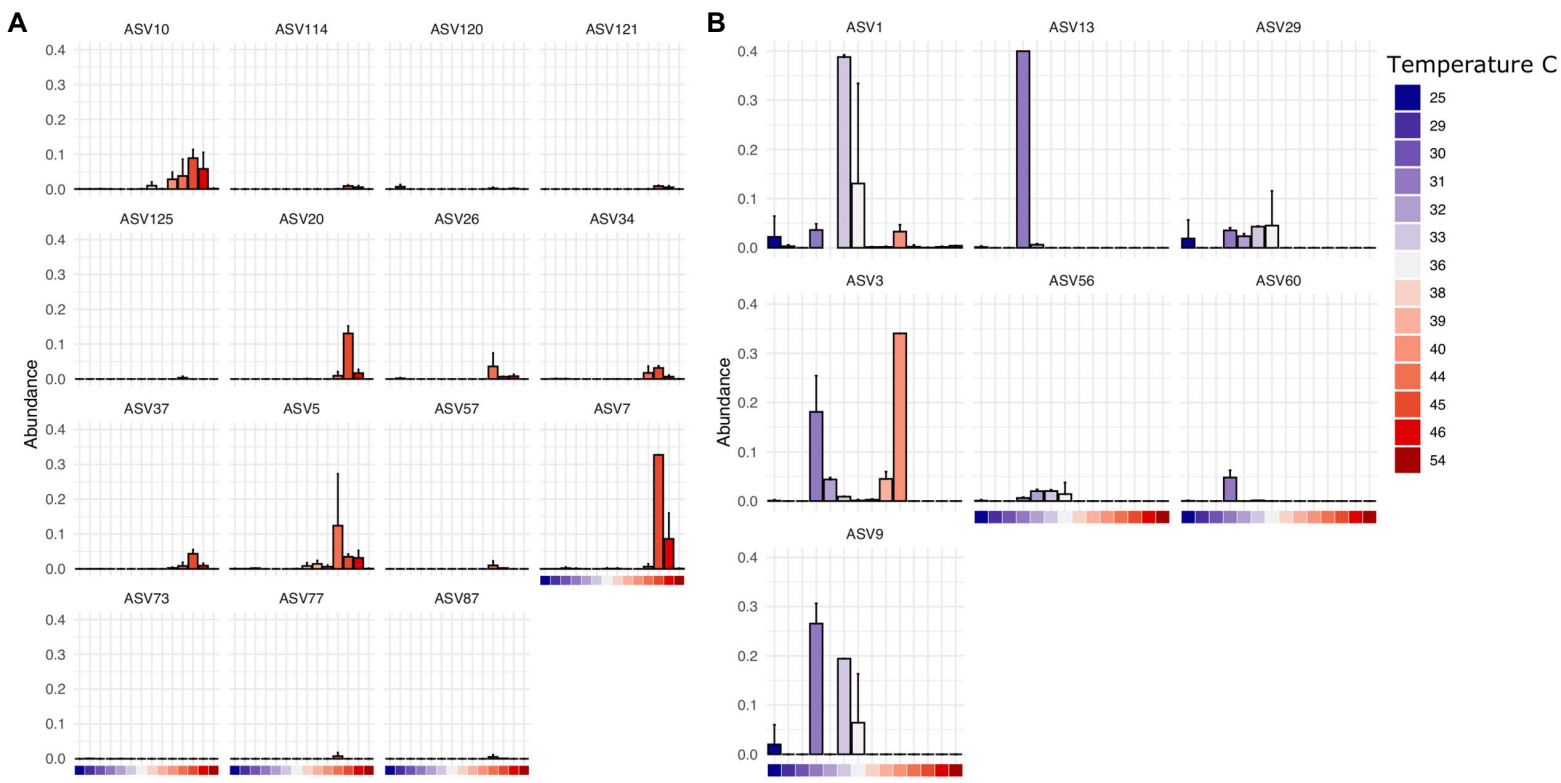
Thermally restricted ASVs in two genera. **(A)** Average relative abundance and standard deviation of the most abundant ASVs assigned to *Clostridium* by temperature at the site of sampling. **(B)** Average relative abundance and standard deviation of the most abundant ASVs assigned to *Ignatzschineria* by temperature at the site of sampling.

Finally, we found that specific bacterial genera had higher or lower relative abundance across different temperature scales, consistent with basic expectations in thermal biology and microbiology research (Zeikus, 1979; Angilletta, 2009). These expectations include decomposition systems (Iancu et al., 2018) where different studies reported groups of decomposer bacteria that exhibited lower limits of ~10°C (Proteobacteria, Actinobacteria), 0°C (Proteobacteria, Firmicutes, Bacteroidetes), and even some Verrucomicrobia able to tolerate temperatures below −10°C. For example, in our results, ASVs assigned to *Clostridium* in this dataset are found at higher abundance at temperatures above 38°C at the site of sampling (Figure 5A) while those assigned to *Ignatzschineria* are typically found at lower temperatures (Figure 5B). Interestingly, some ASVs have a multi-modal distribution across the temperature gradient (e.g., *Ignatzschineria* ASV3). The closest match to this ASV in the NCBI nucleotide database is *Ignatzschineria ureiclastica* or *Ignatzschineria larvae*, both of which are known symbionts isolated from the digestive tract of multiple species of flesh files (Sarcophagidae; Gupta et al., 2011). Interestingly, ASV3 was the taxon predictive of insect association in the random forest models, so the bimodal distribution may be associated with different thermal preferences of winter/summer flies that are in association with the same *Ignatzschineria* taxon. Similarly, while *Ignatzschineria* ASV1 is most highly abundant between 33°C and 36°C, it can also be found at lower abundance at very low temperatures (e.g., 25°C) and higher (e.g., 39°C; Figure 5B).

Given the host of factors associated with PMI estimation, we compared random forest models informed of, or naive to, the factors described above (temperature, BMI, etc) to predict the number of days a donor had been in the field. Our informed model found that PMI explained 25.06% of the total variation with insect presence and temperature at the site of sampling having more predictive importance than any bacterial ASV as measured by the percent increase in MSE. Our naive model explained only 15.62% of the total variation with an ASV assigned to the environmental genera *Gordonia* and insect associated *Ignatzschineria* having the highest predictive power (Supplementary Figure 4). Importantly, the ASV assigned to *Ignatzschineria* (ASV3) is the same that was important for our random forest classification model in discriminating insect presence. Its importance also plummets when insect presence itself is included in the model, suggesting that the metadata observation of insects or the bacterial taxon are useful in identifying decomposition events with and without insects - which could be useful in establishing unobserved insect colonization or confirming when it was suspected. While important in our informed model using other host or environmental factors, it is much less so than when no metadata are considered (Supplemental Figure 4A).

We also applied quantitative PCR (qPCR) to assess the absolute abundance of bacterial taxonomic groups using previously published, taxon-specific primer sets for the phyla Actinobacteria, Firmicutes, Bacteroidetes, and the order Gammaproteobacteria (Yang et al., 2015). Total combined copy numbers detected for the four taxa ranged from 32,714 to 735,150 in summer for samples in the field less than 25 d, and from 2,339 to 29,107 copies for samples in the field from 67 to 218d. The same trend held true for winter samples, with copy number values ranging from 55,572 to 6,305,783 for samples in the field from 12 to 35d, and from 1,144 to 3,253 copies per sample for those in the field from 111 to 272d (Supplementary Table 5). The results support the conclusion that increased time in the field leads to substantially reduced bacterial load over time, possibly from decomposition and subsequent mummification.

Although the semi-quantitative 16S rRNA gene metabarcoding results revealed substantial differences in the relative abundance of ASVs between seasonal samples, we posited that higher taxonomic levels might be more resistant to seasonal variation and thus useful for PMI estimation. To address this, we further assessed the taxon-specific qPCR data for changes in major bacterial populations over time between seasons. In general, over the first 35 days, Firmicutes and Bacteroidetes predominated in the population, with Gammaproteobacteria and Actinobacteria increasing in abundance and dominating at later stages. However, results of qPCR and 16S rRNA sequencing were not in complete agreement, particularly for the Gammaproteobacteria primer set, which appeared to fail in amplification of *Ignatzschineria*-dominated samples associated with winter insects at early time points (Supplementary Figure 6).

## Discussion

Results from this study illustrate that while the bacterial community may provide some information on the PMI and other factors of importance for forensic science, disentangling the impact of these factors and other environmental, individual, and contextual effects remains an ongoing challenge. In the current study we found that many correlated factors, including the temperature of the site of sampling, the presence of insects, decedent BMI, and sex influence the microbial community to varying degrees in different seasons, with temperature and insect presence having the largest overall effect on the community structure in the summer and winter, respectively. Importantly, however, multiple samples from the same individual at the same sampling site may result in substantial differences in the observed microbial community. While the extent of this effect is moderately correlated to the length of time that the individual had been in the environment, it may also be a signal of the degree of mummification of the nares in later stages of decomposition and reduced community cohesion at later time points (Supplementary Figure 6). Moreover, any diagnostic community level patterns are likely to be concealed in individuals with active insect activity. In the current study, insect-associated bacteria dominated the observed microbial community in individuals with active insect activity at the site of sampling, an effect that is more profound in the winter where insect presence accounts for nearly 50% of all of the observed variation in the microbial communities. The homogenizing effect of insects on the microbial community can also be seen in significantly lower beta dispersal in individuals (*p* = 0.001) with active insect activity, even if the insect activity is not at the site of sampling (Supplementary Figure 7). One *Ignatzschineria* taxon (ASV3) is highly predictive of this condition.

Beyond shifts in diversity at the community level, the effects of thermal variation among the different species in a microbiome suggests the need for more detailed information regarding the biology of (at least) the most important marker taxa. To highlight this concept, it is worth considering how thermally adjusted estimates of insect age are implemented in forensic entomology. In that discipline, when conducted optimally (there is heterogeneity in knowledge for all forensically important taxa), reference data are collected on the development of a single species across a range of temperatures (e.g., Grzywacz, 2019). From the thermal response curve, a lower thermal limit can be extrapolated for a linear accumulated degree model or a curvilinear relationship between temperature and development time can be estimated. Therefore, when that species is collected as evidence investigators know if the ambient conditions of the case line up with linear expectations of growth with respect to temperature and if case conditions may require either a curvilinear model or if estimates should not be made with the evidentiary species because linear assumptions are violated. In extreme cases, temperatures may have exceeded or been below the thermal range that supports the biology of the insect in question (see Wells, 2019 and Rusch et al., 2020). Thus, in some cases where an insect taxon is present an estimate of age can be made with a linear accumulated degree model that adjusts for ambient temperature, while in other cases a curvilinear model may be more appropriate, and in others extreme caution should be taken because the thermal limits of the organism in question have been encountered with unknown impacts on predictive ability. In this way *species-specific estimates* of insect age are made with *species-specific development data* and are considered limited by the assumptions made in the analysis (Tarone and Sanford, 2017). However, this strategy is not how thermally adjusted estimates are made in forensic microbiology, as noted above. Limited information regarding when assumptions of forensic microbiology are appropriate for casework restricts the ability to extrapolate from databases to casework and predicts that error in PMI estimates with microbiology will persist until such an accounting of these factors can be made. Such an accounting will require knowledge of the taxa restricted by such environmental factors.

In summary, while the microbial community may be informative of several processes of interest to forensic sciences, in practice many of these patterns may be obfuscated or skewed by a variety of (often correlated) factors including the local environment (e.g., temperature at the site of sampling, presence of insects) or individual dynamics (e.g., sex or BMI). Given these complicating factors, some have advocated for the evaluation of community-level metrics to avoid some of these issues in the context of target taxon analyzes. However, this work indicates that these diversity metrics are not free of potential bias as the presence of insects has its own effects on community diversity. Thus, several aspects of this type of endeavor still need to be assessed. It remains unclear whether community or species level evaluations (or both, such as including *Ignatzschineria* to determine probability of insect presence, and diversity to evaluate condition specific decomposition) of post-mortem remains will be most informative in casework and activity in this area should be continued. However, a more rarely considered dimension to this kind of work (at either the community or species levels) is that of mechanism. The picture painted by this, and other similar projects, indicates that future work should seek to more clearly understand the mechanisms underpinning microbial decomposition of human and animal remains, such as thermal restrictions on microbial taxa, in order to most effectively limit bias from assessments of microbial evidence in forensic casework.

## Data availability statement

The datasets presented in this study can be found in online repositories. The names of the repository/repositories and accession number(s) can be found at: https://www.ebi.ac.uk/ena/browser/view/prjeb57322.

## Author contributions

MA and AT designed the research. ElM, EdM, RZ, TR, and YZ performed the experiments. AM, EdM, YZ, MA, and AT analyzed the data. MA and AT provided materials and resources. AT, AM, ElM, MA, and EdM wrote the manuscript text, with input from the other co-authors. All authors contributed to the article and approved the submitted version.

## Funding

Funding for this project was partially provided by grants from the NSF Center for Advanced Research in Forensic Science (CARFS, #1740434) to MA and AT. Partial support for AT and TR came from NIJ grant 2016-DN-BX-0204.

## Conflict of interest

The Center for Advanced Research in Forensic Science (CARFS) is an NSF/NIJ funded center that includes corporate memberships. During the time of funding of this project, MP Biomedicals, LLC was a member, and is also the manufacturer of the DNA isolation kits and consumables used in this study. Mention of trade names or commercial products in this publication is solely for the purpose of providing specific information and does not imply recommendation or endorsement by the U.S. Department of Agriculture. USDA is an equal opportunity provider and employer. The opinions expressed here do not necessarily reflect those of the National Institute of Justice or the Department of Justice.

The authors declare that the research was conducted in the absence of any commercial or financial relationships that could be construed as a potential conflict of interest.

## Publisher’s note

All claims expressed in this article are solely those of the authors and do not necessarily represent those of their affiliated organizations, or those of the publisher, the editors and the reviewers. Any product that may be evaluated in this article, or claim that may be made by its manufacturer, is not guaranteed or endorsed by the publisher.

## Supplementary material

Supplementary Figure 1 Shared ASVs across season and insect presence or absence. **(A)** Upset plot of the number of shared ASVs between individuals sampled in the summer versus the winter. Few ASVs are shared across groups with the majority (78 and 79%, respectively) only found in one season group. **(B)** Upset plot of the number of shared ASVs between individuals with insect activity at the point of sampling (y), those with insect activity elsewhere on the body (p) and those without insect activity (n). Individuals with no insect activity have a much higher proportion of unique ASVs, indicating the presence of insects has a dampening effect on overall diversity.

Supplementary Figure 2 No significant difference between alpha diversity across season or insect presence. **(A)** Observed number of ASVs and Shannon diversity in summer and winter samples. **(B)** Observed number of ASVs and Shannon diversity in samples with insect activity at the site of sampling, insect activity elsewhere on the body, or no insect activity.

Supplementary Figure 3 Correlation between days in field and maximum distance between swabs from the same individual. Correlation between the maximum distance between beta diversity metrics from different swabs from the same individual. Maximum distance is defined as the difference of the minimum and maximum beta diversity score within a single individual. Individuals who had been placed in the field for a longer time had higher maximum distance than those that were more recently placed in the field (*R*^2^ = 0.65, *p* = 0.004).

Supplementary Figure 4 **(A)** Factors contributing the highest percent increase of MSE in a metadata informed random forest regression model for PMI. **(B)** Bacterial taxa contributing the highest percent increase of MSE in a metadata naive random forest regression model for PMI. Red dots highlight metadata variables of importance, black dots are ASVs found in both models.

Supplementary Figure 5 Lower beta dispersal in individuals with active insect activity. Individuals with no active insect activity have higher median beta dispersal than those with active insect activity (*p* = 0.001). n, no insect activity, p, presence of insects on the remains but not at sample site, y, insects present at sample site.

Supplementary Figure 6 Increased alpha diversity and decreased community cohesion in later stages of decomposition. **(A)** Increase in alpha diversity (Observed ASVs & Shannon diversity) over time since placement in the field. **(B)** Changes in beta diversity by the number of days since placement in the field.

Supplementary Figure 7 Percent abundance results of four phyla interrogated using qPCR and sequencing in two seasons. The relative abundance of Actinobacteria, Bacteroidetes, Firmicutes, and Gammaproteobacteria were measured at different times of decomposition using sequencing and qPCR. Results were compared for concordance and ability to determine post-mortem interval in summer and winter samples. Using qPCR, PMI is best identified using presence of Firmicutes and Bacteroidetes as detectors of early timepoints, and Gammaproteobacteria and Actinobacteria and indicators of later timepoints. Results between sequencing and qPCR varied in the winter samples due to the qPCR’s inability to detect early presence of Gammaproteobacteria.
