# Supplementary figures and images for: The devil is in the details: Variable impacts of season, BMI, sampling site temperature, and presence of insects on the post-mortem microbiome
Source: Front Microbiol. 2022 Dec 7;13:1064904. doi: 10.3389/fmicb.2022.1064904 (PMC9768039; doi:10.3389/fmicb.2022.1064904)

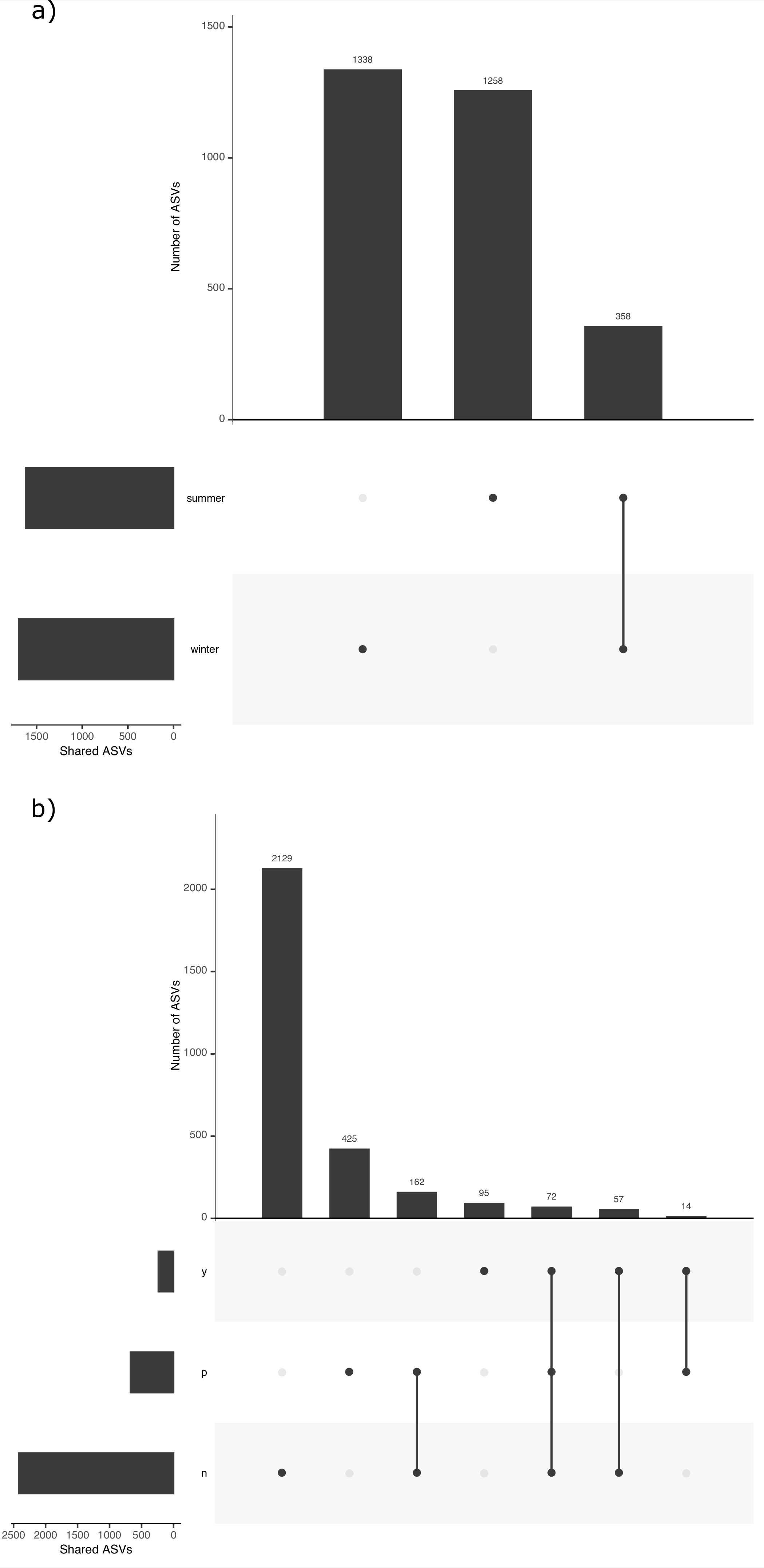

Supplement: Supplementary Figure 1 — Shared ASVs across season and insect presence or absence. (A) Upset plot of the number of shared ASVs between individuals sampled in the summer versus the winter. Few ASVs are shared across groups with the majority (78 and 79%, respectively) only found in one season group. (B) Upset plot of the number of shared ASVs between individuals with insect activity at the point of sampling (y), those with insect activity elsewhere on the body (p) and those without insect activity (n). Individuals with no insect activity have a much higher proportion of unique ASVs, indicating the presence of insects has a dampening effect on overall diversity. [file Image_1.JPEG]

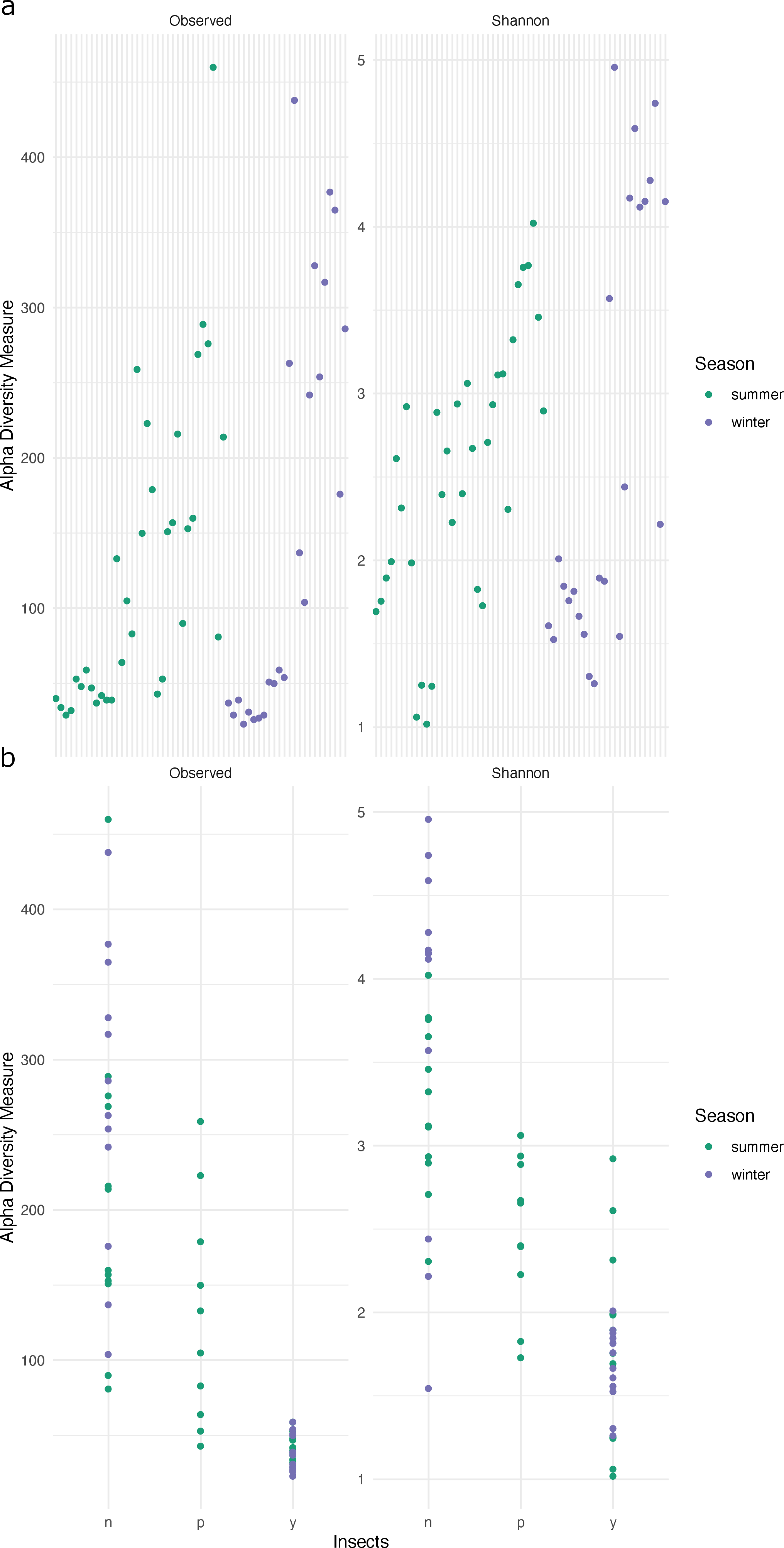

Supplement: Supplementary Figure 2 — No significant difference between alpha diversity across season or insect presence. (A) Observed number of ASVs and Shannon diversity in summer and winter samples. (B) Observed number of ASVs and Shannon diversity in samples with insect activity at the site of sampling, insect activity elsewhere on the body, or no insect activity. [file Image_2.JPEG]

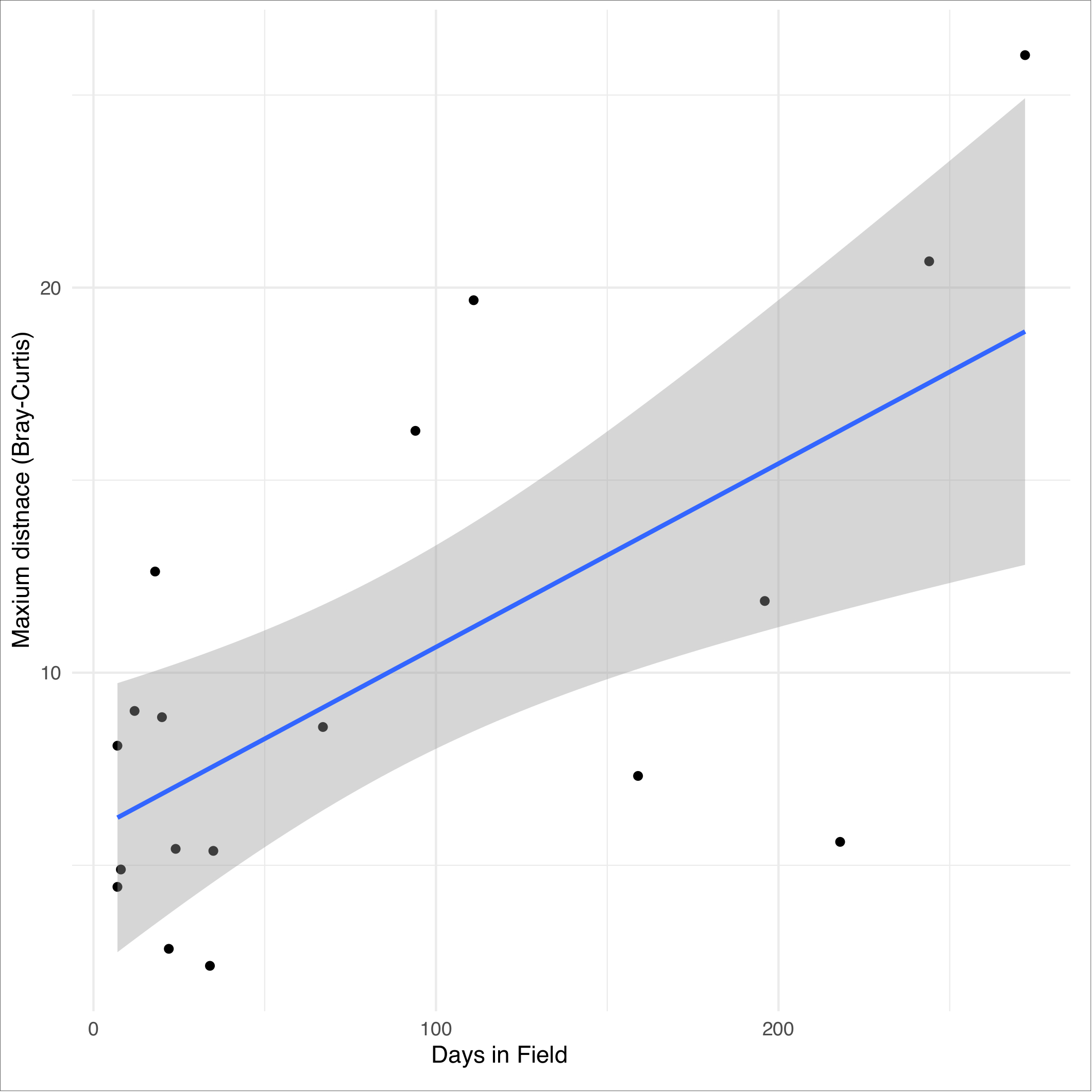

Supplement: Supplementary Figure 3 — Correlation between days in field and maximum distance between swabs from the same individual. Correlation between the maximum distance between beta diversity metrics from different swabs from the same individual. Maximum distance is defined as the difference of the minimum and maximum beta diversity score within a single individual. Individuals who had been placed in the field for a longer time had higher maximum distance than those that were more recently placed in the field (R2 = 0.65, p = 0.004). [file Image_3.JPEG]

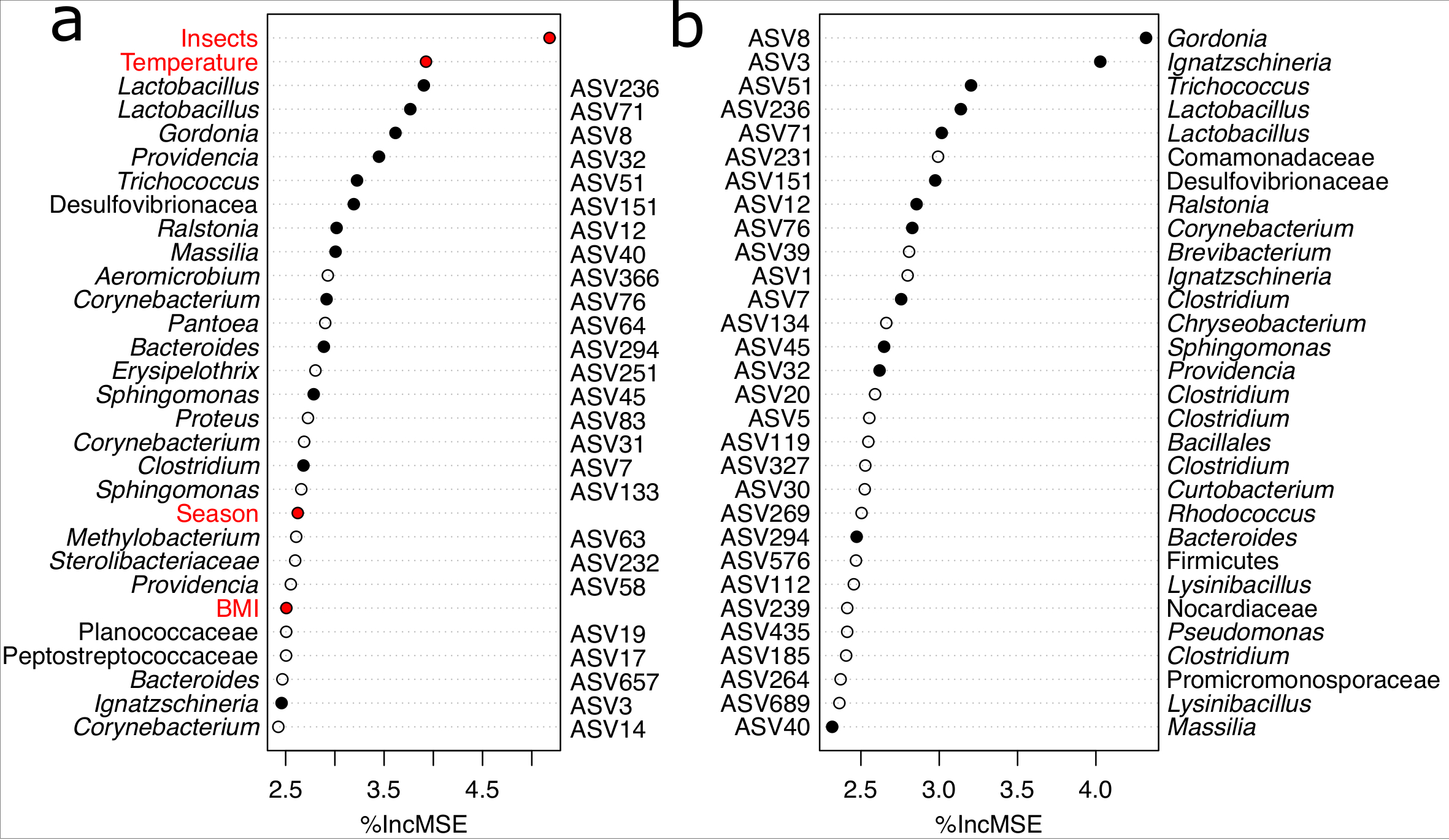

Supplement: Supplementary Figure 4 — (A) Factors contributing the highest percent increase of MSE in a metadata informed random forest regression model for PMI. (B) Bacterial taxa contributing the highest percent increase of MSE in a metadata naive random forest regression model for PMI. Red dots highlight metadata variables of importance, black dots are ASVs found in both models. [file Image_4.JPEG]

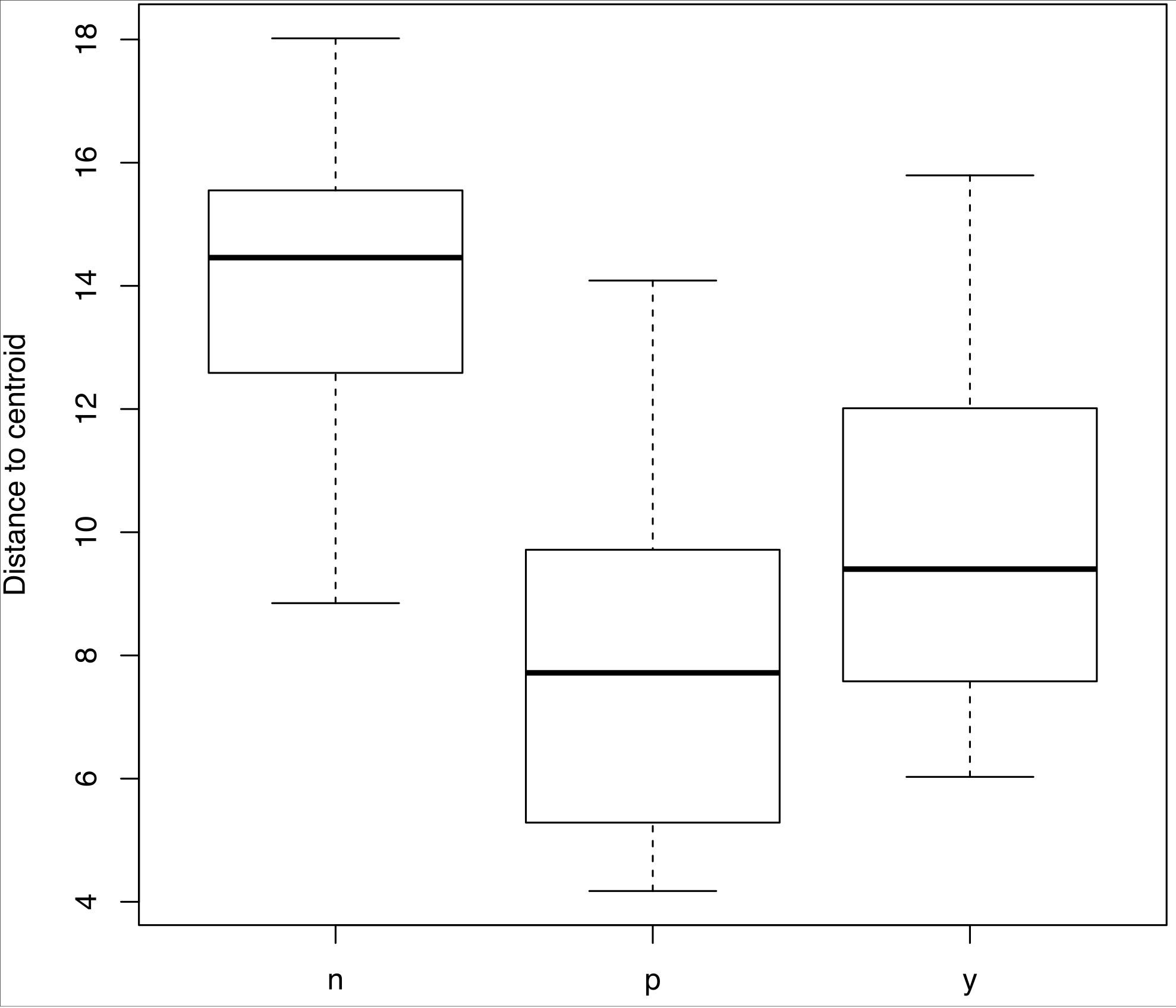

Supplement: Supplementary Figure 5 — Lower beta dispersal in individuals with active insect activity. Individuals with no active insect activity have higher median beta dispersal than those with active insect activity (p = 0.001). n, no insect activity, p, presence of insects on the remains but not at sample site, y, insects present at sample site. [file Image_5.JPEG]

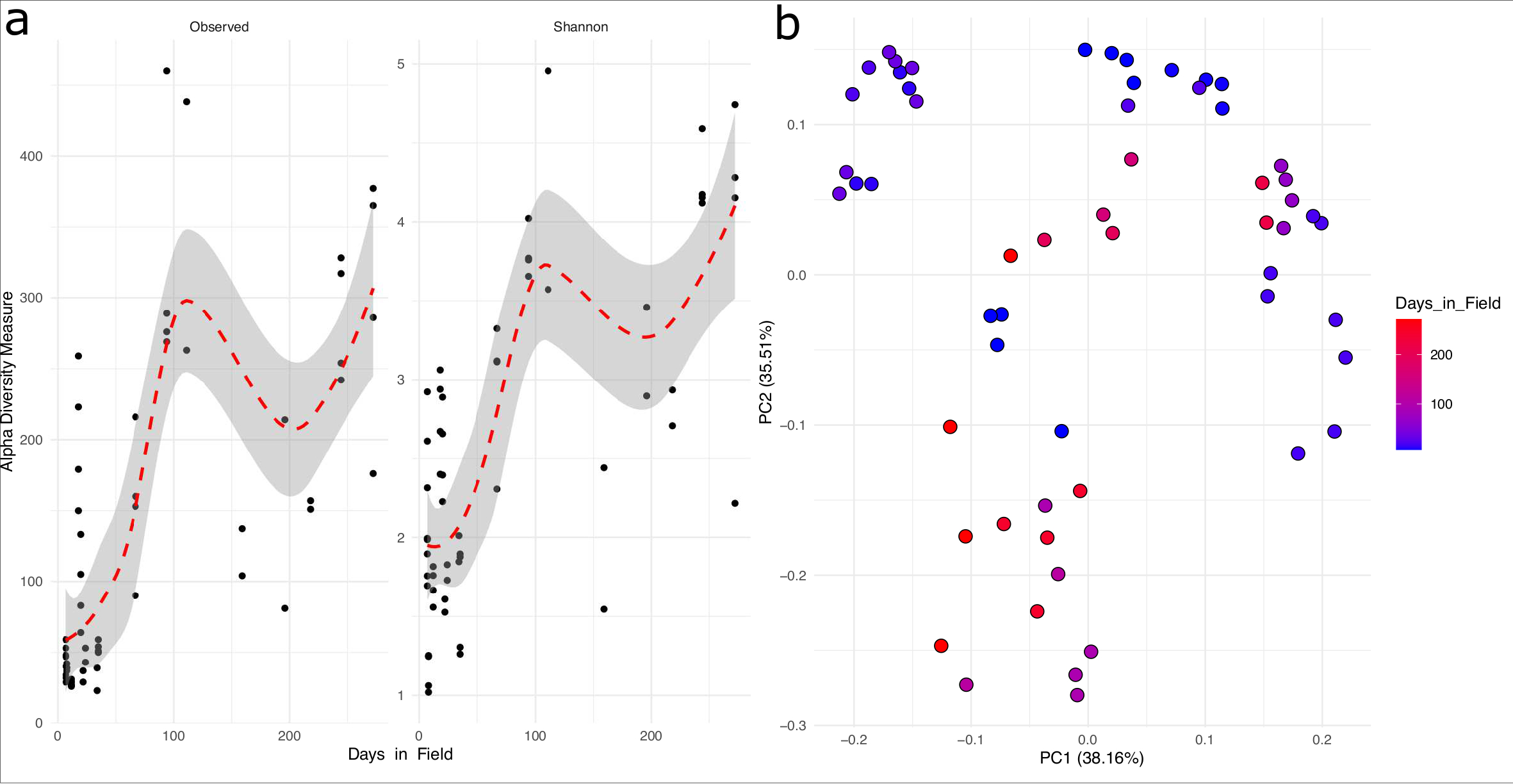

Supplement: Supplementary Figure 6 — Increased alpha diversity and decreased community cohesion in later stages of decomposition. (A) Increase in alpha diversity (Observed ASVs & Shannon diversity) over time since placement in the field. (B) Changes in beta diversity by the number of days since placement in the field. [file Image_6.JPEG]

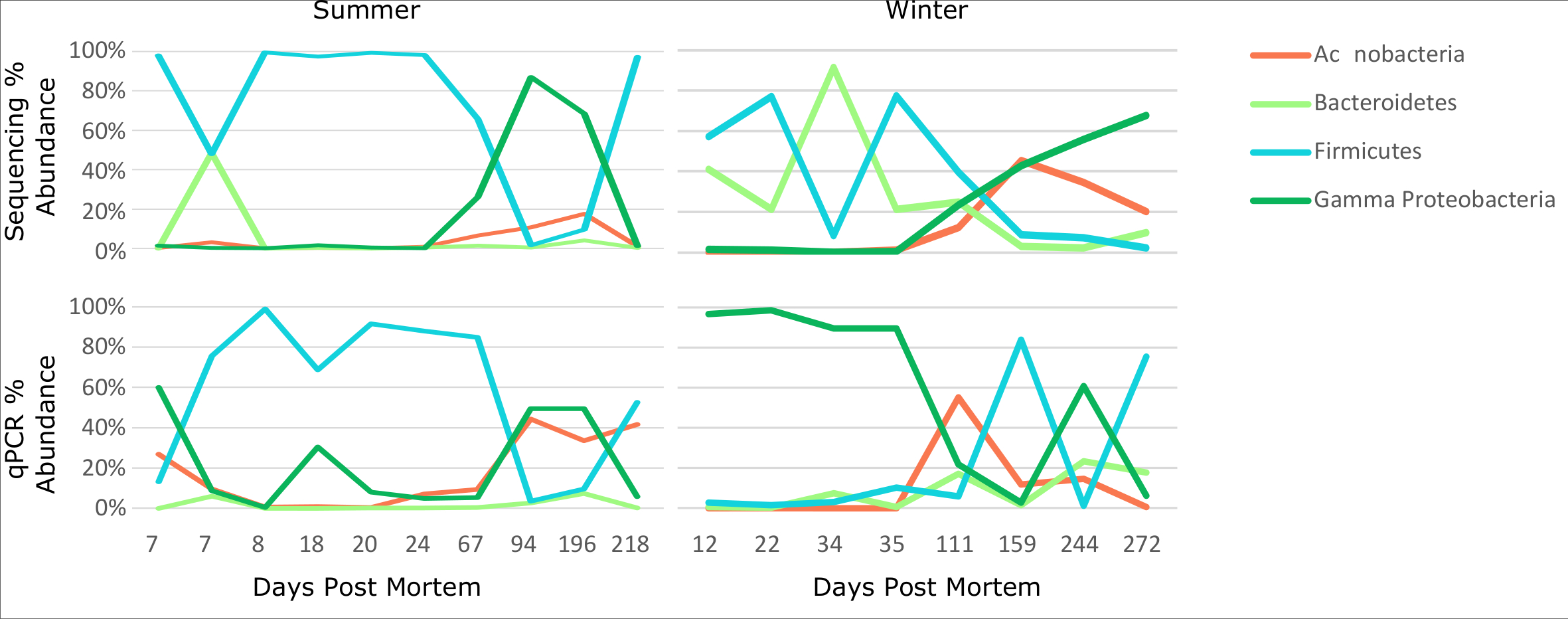

Supplement: Supplementary Figure 7 — Percent abundance results of four phyla interrogated using qPCR and sequencing in two seasons. The relative abundance of Actinobacteria, Bacteroidetes, Firmicutes, and Gammaproteobacteria were measured at different times of decomposition using sequencing and qPCR. Results were compared for concordance and ability to determine post-mortem interval in summer and winter samples. Using qPCR, PMI is best identified using presence of Firmicutes and Bacteroidetes as detectors of early timepoints, and Gammaproteobacteria and Actinobacteria and indicators of later timepoints. Results between sequencing and qPCR varied in the winter samples due to the qPCR’s inability to detect early presence of Gammaproteobacteria. [file Image_7.JPEG]
